# Something old, something new? Herpesvirus genome packaging examined in light of lessons from the tailed bacteriophages

**DOI:** 10.1128/jvi.01785-25

**Published:** 2026-07-06

**Authors:** Laura M. Meißner, Jamie V. Shiah, Olaide A. Adeyemi, Elizabeth J. Bailey, Sara Gelles-Watnick, Allison L. Didychuk

**Affiliations:** 1 Department of Molecular Biophysics and Biochemistry, Yale University 5755, New Haven, Connecticut, USA; 2 Department of Chemistry, Yale University 5755, New Haven, Connecticut, USA; Indiana University Bloomington, Bloomington, Indiana, USA

**Keywords:** viral genome packaging, herpesvirus, tailed bacteriophages, TerL, TerS, terminase

## Abstract

What are viruses, if not nucleic acids surrounded by proteinaceous shells? While the energy-dependent viral packaging motors of tailed bacteriophages have been studied in great depth, our understanding of how eukaryotic double-stranded DNA (dsDNA) viruses package their genomes into nascent capsids remains incomplete. Herpesviruses are ubiquitous human pathogens that drive diverse clinical symptoms. The success of letermovir, a first-in-class human cytomegalovirus genome packaging inhibitor, suggests that this critical step in the virus life cycle can be targeted in other herpesvirus infections. Here, we integrate recent structural, biophysical, and genetic information from representative dsDNA viruses to define the overarching principles of viral genome packaging and highlight evolutionary distinctions. What lessons can we learn from bacteriophages to further our understanding of herpesvirus packaging, and can these insights generate new opportunities for antiherpesvirus therapeutics?

## AN ANCIENT STRATEGY TO PROTECT AND CONDENSE DOUBLE-STRANDED DNA

All viruses must ensure their genetic material can be transferred to a new cell. Packaging the viral genome into a proteinaceous capsid shell protects the genetic material from nucleases and immune recognition. Even the smallest viruses and virophages dedicate a significant fraction of their coding capacity to encapsidation (1, 2). Viral genome packaging strategies can be categorized based on their dependency on chemical energy from ATP hydrolysis (3). Smaller viruses tend to encode capsid proteins that condense and self-assemble around the genome. Larger viruses, particularly those with double-stranded DNA (dsDNA) genomes, undertake a different approach dependent on chemical energy, as they must overcome multiple energetic barriers: the repulsive electrostatics of negatively charged dsDNA, entropy loss from confining dsDNA to a small volume, and the bending energy of dsDNA.

The *Duplodnaviria* are dsDNA viruses that infect all three domains of life (4, 5). This realm is classified by the similarity of core “morphogenetic” genes that allow for the protection and transfer of the viral genome to a new host (5). Duplodnaviruses likely infected the last universal common ancestor (6), and their continued widespread distribution highlights the success of their morphogenetic strategy. The *Duplodnaviria* are classically divided into the *Caudoviricetes*, which are tailed bacteriophages that infect prokaryotes, and the *Herpesvirales*, which infect eukaryotes. (The order *Herpesvirales* includes three families: *Alloherpesviridae*, *Malacoherpesviridae*, and *Orthoherpesviridae*. For simplicity and historical consistency, we refer to the *Orthoherpesviridae* as *Herpesviridae*. The *Herpesviridae* contain the nine human herpesviruses and have been much more extensively studied than the alloherpesviruses and malacoherpesviruses.) A third recently discovered phylum, the *Mirusviricota*, has been tentatively assigned to the *Duplodnaviria* (7). Nine viruses from the *Herpesviridae* family infect humans and cause disease. We discuss herpes simplex virus (HSV-1), human cytomegalovirus (HCMV), and Kaposi’s Sarcoma-associated herpesvirus (KSHV) as representative examples from the alpha-, beta-, and gammaherpesvirus subfamilies. The three herpesvirus subfamilies are separated by millions of years of evolution, resulting in unique strategies for latency and immune evasion and the manifestation of diverse clinical symptoms. However, their viral genome packaging strategies are highly conserved, highlighting this pathway as a potential target for novel, broad-acting therapeutics.

**Fig 1:**
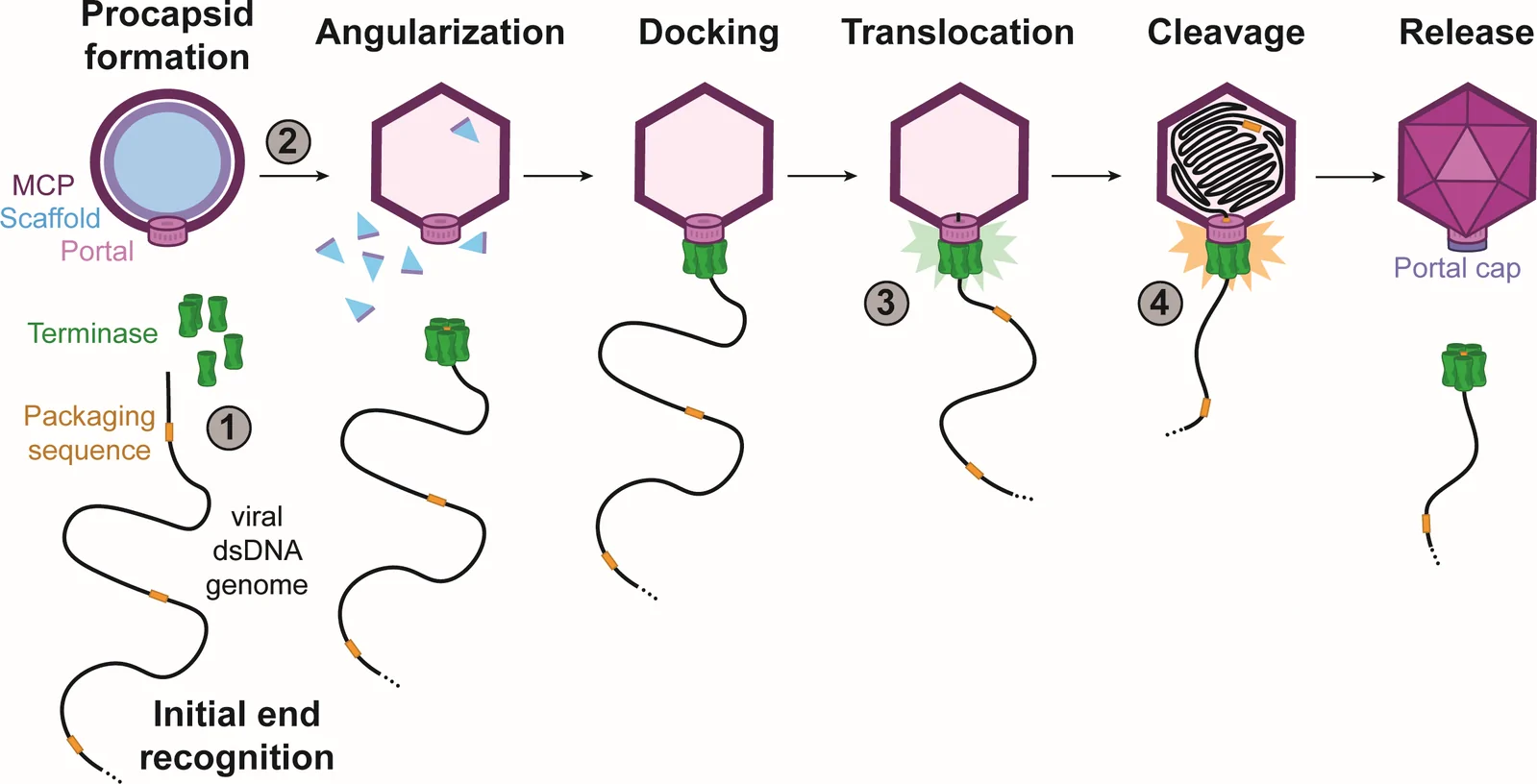
Overview of the herpesvirus capsid assembly and packaging pathway. Spherical procapsids minimally composed of MCP (purple), scaffold (blue), and portal (lavender) forms in the nucleus of infected cells. The scaffold is cleaved by the maturational protease, allowing for capsid angularization; the precise timing of scaffold cleavage relative to initiation of viral genome packaging is unclear. The terminase (green) must recognize the packaging sequence (yellow) in the viral genome, then dock on the portal. The terminase translocates the viral genome into the capsid using energy from ATP hydrolysis. Upon completion of packaging, the terminase cleaves within the packaging sequence, after which the terminase departs and the portal cap binds to stabilize the packaged genome. It is unclear (1) how the initial packaging site is selected, (2) what triggers capsid angularization, (3) how the terminase translocates the viral genome into the capsid, and (4) how headful signals are transmitted from the capsid to trigger cleavage within the packaging site.

Decades of molecular genetics have defined the essential factors required for duplodnavirus packaging (Table 1). Loss of these genes results in accumulation of unpackaged capsids with minimal impact on viral genome replication or the formation of empty icosahedral capsids. All duplodnaviruses encode a powerful molecular motor called the “terminase” to overcome the biophysical challenges of packaging dsDNA. The terminase harnesses chemical energy from ATP hydrolysis to drive conformational changes that translocate the genome into the capsid. The terminase must recognize the viral genome, dock on the capsid at a specialized vertex called the portal, package the viral genome into the capsid, and release the packaged capsid (Fig. 1). In most duplodnaviruses, including herpesviruses and bacteriophage T4, viral genome replication results in concatemers of unit-length genomes separated by a packaging sequence. In such viruses, the terminase is also responsible for dsDNA cleavage to separate the remaining genomic concatemer and complete packaging.

**TABLE 1:** Essential genes for viral genome packaging^*a*^

Component	Caudoviruses					Component	Herpesviruses		
	Φ29	HK97	P22	λ	T4		HSV-1	HCMV	KSHV
TerL	gp16*	gp2*^	gp2*^	gpA*^	gp17*^	Terminase	UL15*^ UL28 UL33	UL89*^ UL56 UL51	ORF29*^ ORF7 ORF67A
TerS	n/a	gp1	gp3	gpNu1	gp16				
Portal	gp10	gp3	gp1	gpB	gp20	Portal	UL6	UL104	ORF43
Other required factors	pRNA, gp3 (terminal protein)	gp74 (HNH)		gpFI, IHF	gp49	Other required factors	UL32, UL17 (CATC)	UL52, UL93 (CATC)	ORF68, ORF32 (CATC)

^*a*^ The protein products conferring ATPase activity (*) and endonuclease activity (^) are indicated. The genes listed in this table are required for packaging during infection. In herpesviruses, loss of these proteins results in the accumulation of empty “B” capsids.

Defined *in vitro* systems have been established for morphologically distinct caudoviruses representative of the myovirus, siphovirus, and podovirus groups, including the *Escherichia coli* bacteriophages T4, λ, and HK97 and the *Bacillus subtilis* bacteriophage Φ29. (The caudoviruses were previously classified into the myovirus, siphovirus, and podovirus families, with divisions based on distinct tail morphology. The order *Caudovirales* and the *Podoviridae*, *Siphoviridae*, and *Myoviridae* families were abolished in 2022 by the International Committee on the Taxonomy of Viruses [8]). By contrast, we lack a molecular understanding of packaging in any herpesvirus because of the challenges of reconstituting a more complex core set of packaging components. Here, we summarize the knowns, unknowns, and path to defining the molecular underpinnings of herpesvirus genome packaging.

## THE CAPSID: AN EMPTY ICOSAHEDRAL PROTEIN SHELL READY FOR PACKAGING

In all duplodnaviruses, capsid shells are assembled prior to packaging, requiring the terminase to funnel the viral genome into the capsid. The physical restraints imposed by compressing a large, dsDNA viral genome into a pre-formed capsid likely constrained the evolution of the packaging machinery. Indeed, early structural work identified a common “HK97” fold in diverse viral capsids and suggested a shared evolutionary history of the caudoviruses and herpesviruses (9–12).

Duplodnavirus capsids are minimally assembled from major capsid protein (MCP), scaffold, and portal. MCP and scaffold assemble into roughly spherical “procapsids” that lack true icosahedral symmetry axes, with outer and inner shells composed of MCP and scaffold, respectively (13–15). Herpesviruses, like many bacteriophages, express a maturational protease fused to the scaffold that self-cleaves between the protease and scaffold domains and then cleaves at the C-terminal end of the scaffold, releasing the majority of scaffold from MCP (16–21). The distinctive shape of the mature capsid is achieved when the scaffold is released, allowing the spherical procapsid to angularize. In some bacteriophages, capsid maturation is coupled to DNA packaging (22, 23). In the mature capsid, MCPs are arranged in the hexons and pentons that form the icosahedral faces and vertices (Fig. 1) (24–26). The timing and trigger for scaffold cleavage and the structural changes that drive angularization remain an open area of inquiry.

Viral genome packaging requires the incorporation of a portal through which dsDNA can enter and exit. The portal replaces an MCP penton at one unique fivefold vertex of the capsid. The portal vertex contains 12 copies of the portal protein, creating a symmetry mismatch (12-fold portal at a 5-fold vertex) (27–30). Despite low or undetectable sequence homology across the duplodnaviruses, portals are structurally similar (12, 31). The portal serves several roles: it engages the terminase, serves as the gateway for dsDNA to enter and exit the capsid, and may nucleate capsid assembly (30, 32, 33).

Herpesvirus capsids are larger in diameter (*T* = 16, ~125 nm) than most of the well-studied tailed bacteriophage capsids and more complex, as their outer shell incorporates additional stabilizing proteins, including the heterotrimeric triplex components and a small capsid protein (34). Subfamily-specific auxiliary proteins, collectively referred to as the capsid-associated tegument complex (CATC), decorate the vertices of the icosahedral capsid and are thought to stabilize the pressurized, dsDNA-filled capsid (35). Herpesvirus capsid assembly is further complicated by the compartmentalization of eukaryotic cells. Capsid proteins are translated in the cytoplasm, but capsid assembly and genome packaging occur in nuclear replication compartments (36–39). Assembly and maturation of herpesvirus capsids have been reviewed in detail elsewhere (34, 35). The greater size and complexity of herpesvirus capsids pose a significant barrier to the establishment of a biochemically defined packaging system, and many questions remain regarding the regulation and order of herpesvirus capsid assembly.

## THE TERMINASE MOTOR: A CONSERVED, POWERFUL MOLECULAR MACHINE

Similarities between bacteriophage and herpesvirus packaging machinery extend beyond the capsid itself. While the primary sequences of MCP, scaffold, and portal are poorly conserved, the “large” terminase gene, TerL, shares sequence similarity across the *Duplodnaviria*. Indeed, sequence homology between putative TerL genes in channel catfish herpesvirus and bacteriophage T4 was an early indication of their evolutionary relationship (40).

In most bacteriophages and all herpesviruses, the product of viral genome replication is a concatemer containing multiple unit-length genomes. The terminase is thus responsible for two activities: (i) converting the chemical energy from ATP hydrolysis into mechanical work to translocate the genome through the portal and (ii) cleaving the packaged dsDNA to release the remaining genomic concatemer. All members of the *Herpesvirales* encode a TerL-like protein containing both ATPase and endonuclease domains (41). The TerL N-terminal ATPase domain drives packaging, while its C-terminal nuclease domain cleaves the genome. The ATPase domain contains classic Walker A and B motifs important for phosphate binding and metal ion coordination (36, 42). The nuclease domain exhibits a classic RNase H-like fold (43–45). The structure and activity of TerL are the focus of a recent review by Leroux and Teschke (46).

In the *Herpesviridae*, the TerL gene is spliced, with exons containing the ATPase and nuclease domains separated by a ~3.5 kb intron (10, 40, 47). In alloherpesviruses (herpesviruses that infect fish and amphibians) and the recently discovered mirusviruses, TerL genes are encoded across multiple exons and in some cases undergo *trans*-splicing (48). Relatively few herpesvirus genes are constitutively spliced, and the conservation of the TerL gene structure across eukaryotic viruses suggests that the virus or host could post-transcriptionally regulate TerL expression.

Herpesviruses have evolved a more complex terminase architecture, where the TerL homolog (HSV-1 UL15) forms a heterotrimer with two other conserved viral proteins required for genome packaging (HSV-1 UL28 and UL33) (37, 49–52). A structure of the HSV-1 terminase revealed that UL28 forms the central core of the heterotrimer and makes extensive contacts with both UL15 and UL33 (53). In studies of the HCMV terminase, co-expression of the three terminase subunits improved the stability of the individual proteins, suggesting that they may co-fold into an obligate heterotrimer (54). Thus, like in bacteriophages, the herpesviruses encode a dual-function “TerL” protein with ATPase and endonuclease domains. However, the herpesvirus TerL functions with its binding partners; we suggest the use of “terminase” to refer to this complex as a whole.

The structure of the HSV-1 terminase showed that six UL15-UL28-UL33 heterotrimers assemble into a hexameric ring (53). The terminase, like many other AAA+ ATPases, must assemble into a multimeric ring to complete its active site, as an “arginine finger” is supplied by an adjacent copy of UL15 (53, 55). The oligomeric state of the Φ29 motor was debated for many years, with conflicting evidence supporting pentameric and hexameric states (56–61). Recent high-resolution cryo-EM structures have revealed that diverse bacteriophages use a pentameric motor that recognizes the dodecameric portal (62, 63). Future work to clarify the oligomeric state of portal- and dsDNA-engaged herpesvirus terminases will illuminate conservation (or lack thereof) with bacteriophage systems.

In HSV-1 and related alphaherpesviruses, UL15 contains a nuclear import signal (NLS) that controls localization of the three terminase subunits to nuclear replication compartments where packaging occurs (37, 64–66). The UL15 NLS interacts with importin α (67). While importin α can accommodate two copies of the UL15 NLS, the position of the NLS makes it unlikely that importin α can simultaneously import two heterotrimers (53, 67). In the betaherpesvirus HCMV, the terminase NLS is present not on the TerL homolog (UL89) but on another terminase subunit, UL56 (67, 68). The UL56 NLS is located on a C-terminal extension predicted to be unstructured (69), potentially allowing for nuclear import of multiple heterotrimers. In gammaherpesviruses, no canonical NLS sequence has been identified in any of the terminase subunits, but a partial NLS sequence present between subunits has been noted (67). It is unclear why terminase nuclear import has diverged between the subfamilies or if the terminase is transported as a multimer of heterotrimers; these differences could drive kinetic differences in localization and control functional ring assembly (67).

In most bacteriophages, a second “small” terminase (TerS) is required for viral genome packaging. As discussed below, the contribution of TerS to different steps in the packaging pathway varies, but it is generally thought to play a role in the initiation of packaging through recognition of the packaging signal (70, 71). TerL and TerS weakly interact and in most cases cannot be biochemically isolated as a complex (70). One of the non-catalytic subunits of the herpesvirus terminase (HSV-1 UL28) is proposed to function as the herpesvirus TerS based on its ability to bind and cleave dsDNA in the absence of other terminase subunits (72–74). Studies in HCMV suggested that UL56 (UL28 homolog), rather than UL89 (TerL), was responsible for ATPase activity (75, 76) and that UL56 and UL89 independently form ring-like structures (77, 78). However, these early results describing functions for non-TerL subunits must be considered in light of the HSV-1 terminase structure. In particular:

1. The HSV-1 terminase structure showed extensive contacts between subunits, including an intermolecular zinc finger between UL28 and UL33. To function independently, UL28 would need to adopt a different structure.
2. In the HSV-1 terminase structure, UL28 is distal to the central channel and is thus unlikely to contact dsDNA threaded through the center of the ring. UL28 residues 433–477 are unmodeled and could in theory contact dsDNA, but these residues are poorly conserved across homologs. It is unclear how UL28 or its homologs could bind dsDNA when part of the terminase complex.
3. If AlphaFold 3 predictions of the HCMV terminase are accurate and the HCMV heterotrimer assembles into a hexameric ring, the proposed UL56 ATP-binding site (residues 709–716) lies at the interface between UL56 and UL89, with no clear pocket available for ATP. It is unclear how UL56 could perform ATP hydrolysis or how this energy could be used during packaging.

Do UL28 and homologs function as independent “TerS” assemblies, then adopt a different fold when binding to UL15 and UL33? How does the terminase recognize herpesvirus packaging sequences? Further work should examine the precise role of each terminase subunit and define how the functional complex is assembled.

## THE SUBSTRATE: GENOME PACKAGING SIGNALS SELECTED BY TerS

Full-length viral genomes must be selectively recognized and distinguished from host dsDNA. Φ29 has a simple solution: the viral genome is replicated as a single unit-length copy instead of a concatemer, and the terminal protein gp3 is covalently attached to the genome termini, allowing for specific recognition and initiation of packaging (79, 80). However, viral genome replication in many duplodnaviruses, including herpesviruses, does not initiate at a packaging sequence, requiring the generation of an initial free end. Specific “*cos*” (cohesive ends) or “*pac*” (packaging) sequences in the viral genome are recognized during packaging initiation by TerS, with important differences between the two strategies observed during packaging termination. The oligomeric state and mode of dsDNA recognition by TerS are variable, making it unclear if a common strategy for packaging signal recognition is shared across viruses (70).

Bacteriophage λ is a canonical example of *cos*-mediated packaging, where exactly one copy of the genome is packaged per virion (81). The *cos* site spans ~200 bp and contains distinct functional subsites (81, 82). TerS binds cooperatively to *cosB* sequences that flank the cleavage site *cosN* (83, 84). After TerS-mediated recruitment of TerL to the packaging site (85), TerL introduces staggered nicks at *cosN*, generating 12-bp cohesive ends (86). This process proceeds along the concatemer, with each packaging event initiating at the cohesive end generated by the previous cleavage event (87). A recent structure of HK97 TerS bound to *cos* dsDNA revealed how sequence-specific contacts are generated (88). After recognition of a *cos* site in the concatemer, the TerL nuclease domain generates an initial free end. As the motor shifts to translocation, it must undergo a large conformational rearrangement as highlighted in a recent study of the λ TerL/TerS holoenzyme (71).

By contrast, T4 employs *pac*-type headful packaging. TerS recognizes *pac* sequences to initiate packaging from a concatemer (89, 90), but termination is sequence-independent. TerL translocates dsDNA until the capsid reaches capacity, then cleaves the concatemer regardless of the underlying sequence (91). The resulting genomes contain an additional ~3 kb from the next genome in the concatemer, yielding roughly 171 kb of packaged dsDNA (92). Subsequent packaging events initiate at the dsDNA end generated by the previous cleavage rather than at the original *pac* sequences, resulting in packaged genomes that are circularly permuted (93). This terminal redundancy enables homologous recombination via the overlapping ends and allows for concatemer formation upon infection of a new host (94).

Herpesviruses employ a hybrid packaging strategy with elements of both *cos* and *pac* systems. They use *pac*-type sequence elements, but these elements are encoded in repetitive regions at the end of the genome; the precise location of cleavage during termination is thus not entirely dependent on sequence. Herpesvirus genome architecture, including the sequence and structure of repeat elements at the termini of the genome, varies across the family. In KSHV, the viral genome is flanked by many (21–41) copies of the 803-bp terminal repeat, each of which contains the packaging signal (95). HSV-1 and HCMV have terminal and internal repeats that separate the unique long (UL) and unique short (US) portions of the genome. The packaging signals in HSV-1 and HCMV consist of two conserved sequences (*pac1* and *pac2*) contained within the *a* sequence and present internally and at both ends of the genome. The *pac1* element consists of a T-rich region flanked by G-rich sequences, while *pac2* contains a CGCGGCG motif adjacent to a T-rich region; these two conserved sequences drive cleavage at a fixed distance between the sites (96–98) (reviewed in reference 99).

The herpesvirus packaging sequence, given its high GC content, may deviate from canonical B-form DNA. All herpesvirus packaging signals can form G-quadruplex structures, and the ability to form these alternative dsDNA structures is conserved across the family (100). The contribution of host cell factors to *pac* site recognition or herpesvirus genome packaging more broadly remains to be determined (101). Because the *pac* motifs are repeated between the UL and US segments (HSV-1/HCMV) and in the terminal repeats (KSHV), herpesviruses likely use a combination of sequence-dependent recognition and pressure-sensing headful mechanisms to ensure that a full genome is packaged before cleavage of the concatemer. How the first *pac* site for initiation of herpesvirus packaging is selected remains unclear.

## BEYOND THE MOTOR: ACCESSORY FACTORS REQUIRED FOR PACKAGING

Even Φ29, with its small genome and simple viral genome selection strategy, requires additional factors beyond TerL to facilitate packaging. Φ29 employs a packaging RNA (pRNA), a 174-nucleotide RNA essential for motor assembly and selective packaging (9, 102, 103). The stoichiometry of pRNA was debated for years, with biochemical and genetic studies suggestive of a hexameric assembly (56, 58, 104). Structural work has resolved the pRNA as a pentameric ring on the dodecameric portal (57, 105–107), creating a complex symmetry mismatch and creating a binding site for pentameric TerL. The pRNA additionally interacts with the MCP through symmetry-matched contacts (105, 106, 108).

λ gpFI is a packaging chaperone that is essential *in vivo* but non-essential *in vitro*, although its inclusion in *in vitro* packaging systems enhances packaging efficiency ~200-fold (109–112). Rather than participating directly in translocation, gpFI coordinates packaging initiation by bridging the capsid and terminase-DNA complex while also accelerating terminase recycling after cleavage (109, 110, 113, 114). Suppressor mutations in terminase and capsid bypass the requirement for gpFI (114), demonstrating that gpFI supports pre-existing interactions rather than contributing an independent function. Interestingly, a host factor (*E. coli* integration factor IHF) is also important for λ viral genome packaging by stimulating cleavage at the *cos* site (111).

HK97 encodes gp74, an HNH endonuclease that physically interacts with TerL and stimulates terminase-mediated cleavage at the *cos* site (115, 116). Structural studies revealed that gp74 contains an α-helical insertion within the loop region of the HNH fold that regulates divalent metal ion binding, providing a molecular basis for how the protein enhances *cos* site cleavage by the terminase (117). The metal-binding histidine residue in the HNH motif is essential for gp74 function, as mutation of this residue abolishes the ability of gp74 to enhance terminase activity (116). Like λ gpFI, HK97 gp74 is essential for packaging *in vivo* but only enhances *cos* cleavage efficiency *in vitro* (116). Accessory factors like gpFI and gp74 may be required *in vivo* because of low terminase expression, explaining their ability to be bypassed *in vitro* when high concentrations of terminase are used (116).

HNH endonucleases can be found in many bacteriophage genomes, suggesting that they play a conserved role (116). In the circularized HK97 prophage, gp74 is positioned directly adjacent to the terminase genes encoding TerS and TerL. This genomic organization is conserved across diverse bacteriophages (116). A similar genome architecture grouping packaging-related genes is not conserved in herpesviruses, where the three subunits of the terminase (UL15, UL28, and UL33) are not adjacent to each other in the genome. Interestingly, a herpesvirus packaging accessory factor (HSV-1 UL32, discussed below) is positionally conserved and adjacent to the smallest terminase subunit (UL33) in all herpesviruses.

## AN UNKNOWN: THE ROLE OF A HERPESVIRUS PROTEIN REQUIRED FOR PACKAGING

The *Herpesviridae* encode an additional factor required for viral genome packaging (HSV-1 UL32). Loss of this factor results in a packaging-defective phenotype identical to loss of the terminase itself, where viral genome replication proceeds normally but concatemers remain uncleaved and empty capsids accumulate in the nucleus (118–121). Recent structures of the packaging accessory factor from HSV-1, HCMV, EBV, and KSHV reveal a highly conserved core fold and a propensity to oligomerize into pentameric rings (122, 123). Formation of a ring brings together positively charged residues critical for viral genome packaging, although the precise function of this region remains unknown. While it is tempting to propose that the packaging accessory factor binds the viral genome during infection, the central channel of the pentamer is too narrow to accommodate dsDNA.

Recently, HCMV UL52 has been shown to interact directly with terminase subunits UL56 and UL89 and portal protein UL104, potentially physically bridging core components of the packaging machinery (124). Whether these interactions are maintained in other herpesviruses remains to be determined. The conserved architecture and pentameric symmetry of this factor hint at a shared function across the *Herpesviridae*. Do pentameric herpesvirus packaging accessory factors bridge interactions between the terminase and capsid, like Φ29 pRNA? Does this factor aid in substrate selection like bacteriophage TerS? Does its pentameric symmetry allow it to act as a chaperone for the motor to help it assemble into an oligomeric ring? Do eukaryotic-infecting viruses face unappreciated obstacles that this enigmatic packaging accessory factor overcomes?

## BRINGING IT ALL TOGETHER: THE MOLECULAR MECHANISM OF PACKAGING

**Fig 2:**
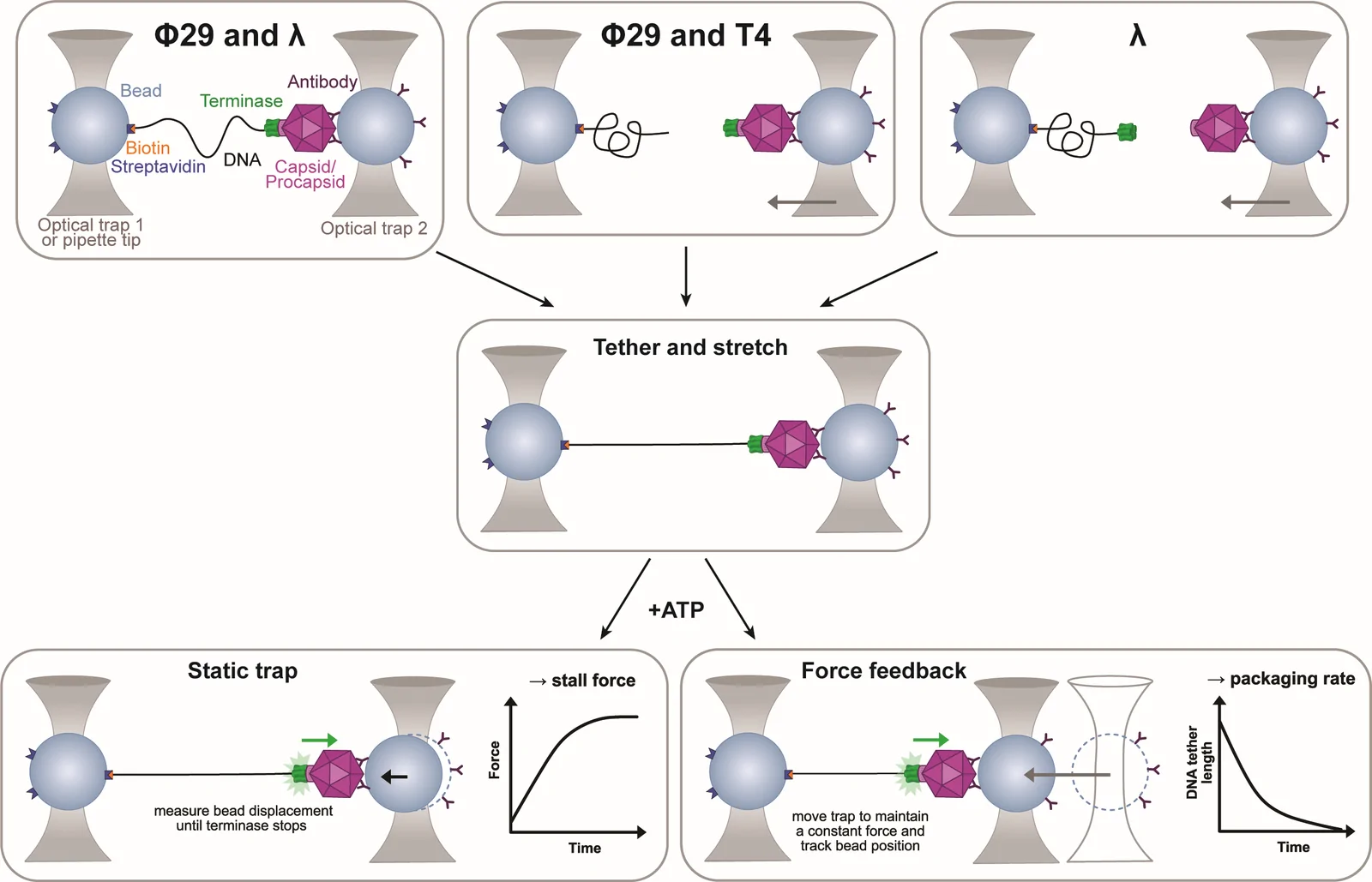
Overview of established single-molecule optical tweezer experiments to study bacteriophage packaging. Functionalized micrometer-sized beads (light blue) are caught with optical traps (gray). Trap 1 can be replaced by a pipette tip. dsDNA (black) functionalized with biotin (orange) is attached to a streptavidin-coated bead (blue). Capsid (purple) is attached to a second bead coated with anticapsid antibody (indigo). In different systems, the terminase (green) is either pre-assembled with the capsid and DNA (Φ29 and λ), pre-bound to the capsid (Φ29 and T4), or pre-bound to the DNA (λ). In the latter two cases, a tether is established by moving the second trap until a DNA-terminase-capsid complex is formed. The tether is stretched, and packaging is initiated through the addition of ATP. Two types of experiments can be performed. In a static trap experiment (lower left), the terminase pulls the bead out of trap 2 (displacement <1 µm) until the motor stalls. Plotting the force (derived from bead displacement) over time yields the stall force. In a force feedback experiment, trap 2 is moved (distance >1 µm) to maintain a constant force (e.g., 5 pN), and the bead position is monitored. The distance between the beads is proportional to the DNA tether length. Plotting tether length over time allows for the calculation of the terminase packaging rate.

*In vitro* packaging systems have been developed for bacteriophages including T4, λ, HK97, and Φ29 (125–129). Bulk solution methods allow for the precise definition of required factors, while single-molecule measurements can paint a detailed picture of the mechanism of dsDNA translocation. Optical tweezers are a powerful system to track the progression of individual packaging events, measure the force generated by the motor, and observe detailed motor behavior including slipping and pausing (Fig. 2). These approaches have highlighted the remarkable force-generating properties of bacteriophage motors (130–132).

The Φ29 terminase translocates dsDNA up to 165 bp/s in a highly processive manner (60, 132, 133). ATP hydrolysis is tightly coordinated between the five motor subunits. The motor cycles between 10-bp “burst” periods where four subunits hydrolyze ATP, release phosphate, and translocate the dsDNA in 2.5-bp steps and a “dwell” phase where ADP is exchanged for ATP without translocation (59, 60, 134–136). The fifth “special” subunit does not translocate dsDNA upon ATP hydrolysis and instead regulates the hydrolysis cycle of the other four subunits (59, 135, 137). The motor pushes dsDNA into the capsid by transitioning between helical and planar conformations, termed the “helical inchworm” model (136, 138). The ATP-bound state of the motor has a higher affinity for dsDNA and binds the helical phosphate backbone (134, 136). ATP hydrolysis weakens dsDNA binding and allows for a return to the planar conformation, thereby translocating dsDNA into the capsid (62, 136, 138). When a single subunit is blocked (e.g., with a non-hydrolysable ATP analog), the motor stalls, emphasizing the tight coordination between subunits (132, 135).

While TerL from T4 and Φ29 are structurally similar, the T4 motor operates in a different manner in which the terminase subunits work through a “continuous burst model” in which the subunits are not as strictly coordinated (139). One subunit hydrolyzes ATP and translocates dsDNA, while the other subunits exchange nucleotides, and the conformation of the motor is constantly remodeled in a “hand-over-hand” fashion (139). The simultaneous firing and recharging of the motor enables average packaging rates of 700 bp/s and a maximum rate of 2,000 bp/s (131, 139). Due to the loose coordination of subunits, the motor tolerates up to three ATPase defective subunits and continues translocation, albeit with slips, pauses, and lower velocity (139). In the paused state, packaged dsDNA exits the capsid, allowing the motor to readjust and circumnavigate obstacles (139, 140). The λ terminase behaves more like the T4 terminase, with an average packaging rate of 600 bp/s, but with overall tighter binding to dsDNA potentially mediated by TerS (131, 141).

Bacteriophage motors can accommodate different substrates but can stall at intercalators, modifications, or deviations of canonical B-form dsDNA. The Φ29 motor adapts to pitches other than that of B-form dsDNA (138). While T4 terminase packages A- and B-form dsDNA with the same velocity, it fails to translocate dsDNA-RNA hybrids or dsRNA (142), and intercalating dyes such as acridine inhibit packaging (143). Interestingly, the T4 motor can reinitiate packaging and package multiple short (<100 bp) fragments into the same capsid (144). This adaptability could help it circumnavigate roadblocks and facilitate coordination of packaging with transcription and recombination (145). The herpesvirus terminase must overcome nicks and gaps in the newly replicated genome (146), suggesting that its motor must also be able to adapt to non-ideal substrates.

Despite differences in genome structure and use of accessory factors across bacteriophages, general principles of viral genome packaging emerge. The motors translocate ~2 bp per ATP molecule (59, 134, 147, 148) with efficiencies comparable to or higher than other molecular motors (132, 149, 150). Intriguingly, the packaging rate is correlated to viral genome size, such that all bacteriophages package their genomes in the span of a few minutes (Table 2). Another common feature is the incredible force output (up to 60 pN), highlighting their status as the most powerful natural motors (130–133). Do herpesvirus terminases generate comparable forces, package their large genome at similar or higher rates, and show mechanistic similarities to the flexible but fast T4 motor? These questions can only be addressed with an *in vitro* herpesvirus packaging system.

**TABLE 2:** Properties of packaging motors and systems^*a*^

Property	Caudoviruses					Herpesvirus
	Φ29	HK97	P22	λ	T4	HSV-1
Packaged genome length (kb)	19.3	39.7	~43	48.5	~172	~125
Termination mechanism	Unit-length genome	*cos*	Headful (*pac* site used for initiation)	*cos*	Headful (*pac* site used for initiation)	Headful (cleavage within *pac* site/terminal repeats)
Bulk genome packaging system?	Yes	Yes	Yes	Yes	Yes	No
TerS required *in vitro* for translocation?	N/A (no TerS) gp3 and pRNA required (132)	Yes (129)	Yes (151)	Yes (131)	No (inhibitory on linear dsDNA) (152)	Unknown (no *in* vitro packaging system)
Single molecule *in vitro* packaging system?	Yes	No	No	Yes	Yes	No
Maximum packaging rate (bp/s)	100–165 (132, 133)			~760 (131)	2,000 (130, 139)	
Average packaging rate (bp/s)	~80 (133)			600 (131)	700 (130, 139)	
Completion time (min)	4			1.3	3–4	
Force (pN)	57 (132)			>50 (131)	>60 (130)	
Pressure in packaged capsid (atm)	59^*b*^ (132)	10^*c*^ (153)	17 (154)	20–25 (155–157)	20–35^*d*^	20 (158)

^*a*^ We include HSV-1 as a representative herpesvirus, given that no *in vitro* system exists for any herpesvirus.

^*b*^ Estimate.

^*c*^ Simulation.

^*d*^ Commonly cited estimate.

A reconstituted herpesvirus packaging system would also be useful to define the minimum components required for motor activity. Substantial differences exist in bacteriophage systems (i.e., the *in vitro* dependency on TerS), potentially stemming from differences in substrate selection and formation of an initiation-competent packaging complex (Table 2). *In vitro* packaging systems typically use linearized dsDNA, in some cases shorter-than-genome length dsDNA, allowing for the termination-related function of TerS to be decoupled from the generation of an initial free end. In λ, TerS may also serve to “catch” the viral genome in the case of TerL slippage on dsDNA, thus contributing an additional role as a sliding clamp (141). In HK97 and P22, TerS stimulates TerL ATPase activity (129, 159). In T4, TerS stimulates the ATPase activity of TerL while inhibiting nuclease activity (160–164). It is unclear how the herpesvirus packaging accessory factor or non-catalytic subunits of the herpesvirus terminase contribute to regulation of ATPase and endonuclease activity.

## UNDER PRESSURE: BIOPHYSICAL CONSTRAINTS OF PACKAGING dsDNA

Regardless of their packaging machinery or mechanism, duplodnaviruses must overcome the same physical barriers inherent to condensing dsDNA. Viral genome packaging is a highly unfavorable process because of electrostatic repulsion from the phosphate backbone, the stiffness of dsDNA, and loss of entropy (165). While some dsDNA viruses (e.g., papillomaviruses) use histones to condense their genome (166, 167), duplodnaviruses package naked dsDNA. Encapsidated dsDNA is considered to be “near-crystalline,” with ~2.6 nm spacing between helices and densities reaching 500 mg/mL (11, 168, 169). The terminase must generate high forces to overcome these energetic barriers and package dsDNA into the capsid, creating stored potential energy that is released upon genome ejection in the next host.

The pressure inside packaged capsids can be measured by triggering genome release under different osmotic conditions, where ejection stops when the internal capsid pressure is equal to the surrounding osmotic pressure (155, 158, 170, 171). Measurements of bacteriophage and herpesvirus capsids revealed internal pressures of 10–59 atm, several times higher than the typical pressure of a car tire (~2 atm) (132, 154–156, 158, 172) (Table 2). Pressure within the capsid can be modulated either by packaging less dsDNA or by electrostatic screening of repulsive charges (i.e., with cations or multivalent amines capable of diffusing through the capsid lattice) (155, 173, 174). Capsid pressure is critical for viral delivery into the next host cell (for bacteriophages) or nuclear pore (for herpesviruses). Reduction of capsid osmotic pressure reduces the efficiency of HSV-1 genome ejection (172, 175). Over-pressurization of the capsid is also deleterious, as packaging even a 10% longer genome greatly reduces infectivity in bacteriophage λ (176). The high capsid internal pressure is necessary not only for ejection but also to confer capsid stability and allow capsids to tolerate external forces (173). Duplodnaviruses have evolved to balance their genome length, motor properties, and capsid stability; they have optimized genome compaction in the face of impressive physical constraints and utilized the resulting pressure to drive genome delivery.

The act of packaging dsDNA changes its conformation, and dsDNA conformation, in turn, affects the biophysics of both translocation and ejection. Electrostatic interactions with the portal stretch and compress dsDNA as it is translocated into the capsid, and these deformations may contribute to translocation (143, 177, 178). The rate of packaging also affects the dsDNA conformation: if the packaging rate is too high, the dsDNA can become kinked (179). As the capsid is filled, the dsDNA exhibits non-equilibrium conformational dynamics with relaxation times of more than 10 min, indicating that both dsDNA conformation and capsid fill level affect the packaging rate (180–182).

Cryo-EM reconstructions of packaged bacteriophage and herpesvirus capsids reveal concentric layers of packaged dsDNA that are highly ordered near the capsid shell but exhibit a disordered central core (11, 28, 183, 184). Early structural studies and simulations suggested that dsDNA is packaged as an inverse spool (185, 186). Recent all-atom molecular dynamics simulations of HK97 packaging instead suggest that dsDNA is threaded into the capsid by a loop extrusion mechanism, generating a unique dsDNA configuration in each packaged capsid (153). The Φ29 terminase rotates dsDNA during translocation, introducing torsional strain (60). Simulations of Φ29 packaging demonstrated that rotation, but not revolution, is necessary for appropriate organization of the encapsidated genome (187). In contrast, a revolution mechanism was proposed for the HSV-1 terminase based on the larger inner channel diameter formed by a hexameric (rather than pentameric) motor (53, 188). It remains to be determined how this proposed herpesvirus packaging mechanism affects dsDNA conformation.

## ALL GOOD THINGS COME TO AN END: COMPLETION OF PACKAGING

In viruses with concatemeric replicated dsDNA, the packaged genome must be separated from the remaining genome through endonucleolytic cleavage by the terminase. The trigger for endonuclease domain engagement is mechanistically distinct in *cos* and *pac* viruses. In *cos* viruses, sequence-specific recognition of a second *cos* site induces cleavage. By contrast, *pac* viruses do not recognize a specific sequence and instead use a pressure-sensitive signal from packaging a “headful” of genome to initiate cleavage. The headful signal is conveyed through the portal, which undergoes a large conformational change as pressure pushes it outward relative to the capsid shell. Studies comparing procapsid, unpackaged, and mature capsid structures in headful bacteriophages and herpesviruses have shown the extent to which the portal is repositioned relative to the outer capsid shell after packaging (189–194). When bound to unpackaged capsids, the active site of the terminase endonuclease domain is thought to be sterically inaccessible; conformational changes in the portal induce movement of the lid away from the endonuclease active site, activating the nuclease (195, 196). Interestingly, recent work in the *cos* virus HK97 suggests that pressure sensing also contributes to TerL dissociation, suggesting that pressure-induced conformational changes may contribute to both *cos* and *pac* termination (63).

After dsDNA is packaged, the capsid undergoes conformational changes to retain the viral genome and associate with additional factors to continue maturation. In bacteriophages, addition of the neck, tail, and/or plug proteins further aids in dsDNA retention (197–199). In herpesviruses, one component of the CATC complex (HSV-1 UL25) adopts a different conformation at the portal vertex, forming a pentamer that acts as a portal cap (27, 28, 200–202). Loss of the portal cap resulted in the accumulation of “A” capsids and cleaved but unpackaged viral genomes, consistent with a model where packaging was initiated but the genome was not successfully packaged within the capsid (193, 203, 204). The timing and coordination of herpesvirus packaging termination and portal cap binding are poorly understood.

## THE GOAL: THERAPEUTIC TARGETING OF HERPESVIRUS GENOME PACKAGING

**Fig. 3:**
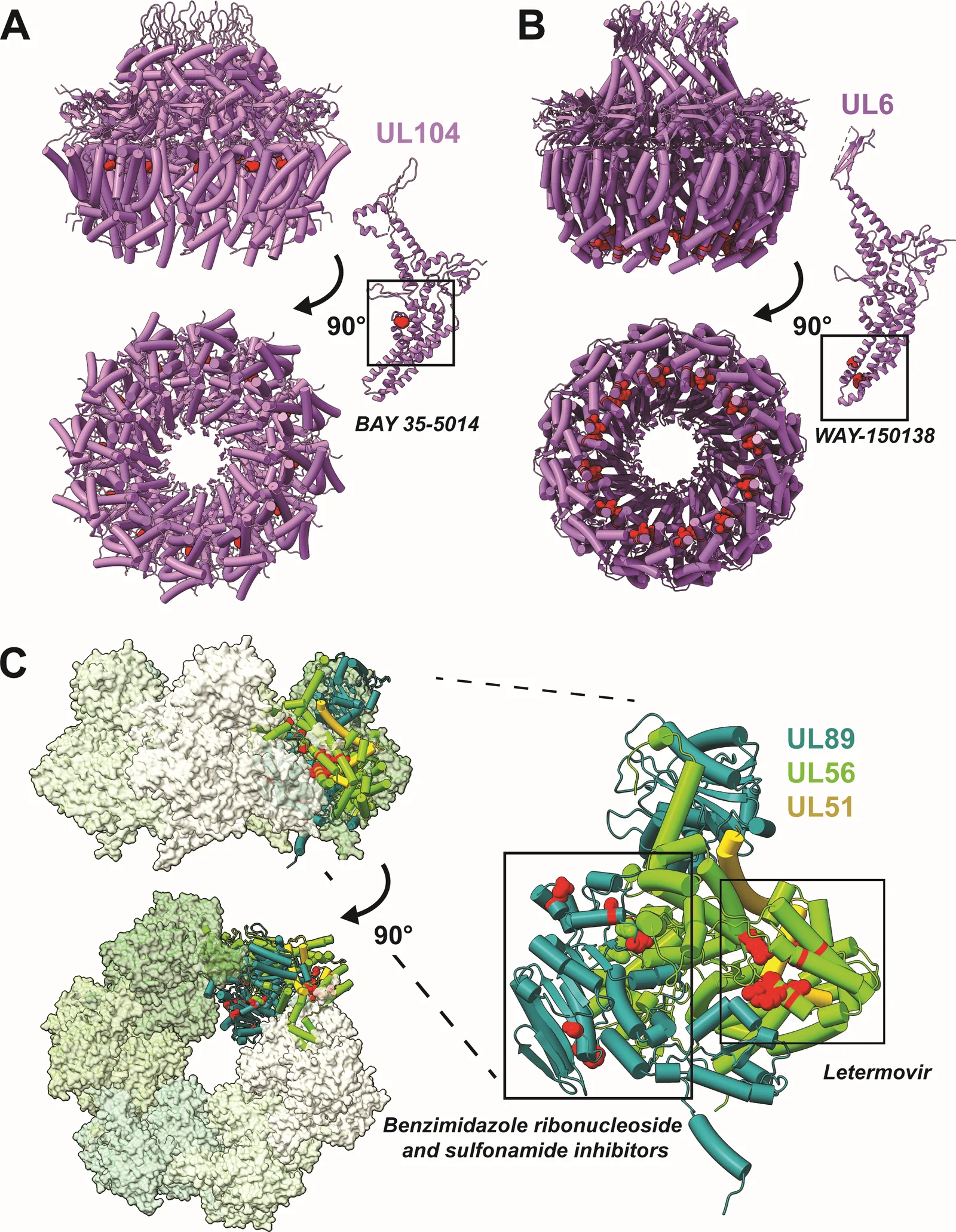
Summary of pre-clinical and clinically used herpesvirus small molecules, indicating the position of resistance mutations. (**A**) Structure of the HCMV portal UL104 (PDB 8HEV). The resistance mutation to BAY 35-5014 is highlighted in red. Resistance mutations to BAY 35-5014 also arise in the HCMV terminase. (**B**) Structure of the HSV-1 portal UL6 (PDB 9OP8). Resistance mutations to WAY-150138 are highlighted in red. (**C**) AlphaFold 3 (69) was used to generate a model of the HCMV terminase protomer, with UL89 shown in teal, UL56 shown in green, and UL51 shown in yellow. N- and C-terminal regions predicted to be unstructured are hidden for clarity. The protomer prediction was docked on the HSV-1 terminase structure (PDB 6M5S) to visualize a hexameric assembly, shown as a surface in shades of green (left). The site of resistance mutations against small molecule inhibitor letermovir, benzimidazole ribonucleoside inhibitors (TCRB and BDCRB), and sulfonamide inhibitors (BAY 38-4766 and BAY 35-5014) are indicated in red.

Herpesvirus antivirals in clinical use largely target a single pathway: viral dsDNA replication. While these drugs (e.g., valacyclovir, valganciclovir, cidofovir, and foscarnet) are potent, their utility is limited by the development of resistance mutations and association with adverse toxicities, highlighting the need for drugs that act through novel mechanisms of action (205, 206). Viral genome packaging is critical for the production of infectious virus and thus holds considerable potential as a therapeutic avenue. Benzimidazole ribosides (2,5,6-trichloro-1-β-D-ribofuranosyl benzimidazole [TCRB] and 2-bromo-5,6-dichloro-1-(β-D-ribofuranosyl) benzimidazole [BDCRB]) and sulfonamides (BAY 38-4766 and BAY 35-5014) inhibited HCMV replication, with resistance mutations mapping to the terminase and portal (207–212) (Fig. 3; Table 3). Thiourea derivatives, one of which was developed into WAY-150138, inhibited HSV-1 replication by acting on the portal protein UL6 (213, 214). However, these early terminase and portal inhibitors are not undergoing further clinical testing (31, 215).

**TABLE 3:** Summary of pre-clinical and clinically used herpesvirus small molecules targeting viral packaging machinery, indicating the position of resistance mutations^*a*^

Name	Proposed target(s)	Antiviral activity	Resistance mutations	References
Letermovir	Terminase	HCMV	UL56-C25F, L51M, V231L*, V231A, N232Y*, V236L, V236M*, V236A, E237D, L241P, T244K, L257F*, L257I, Q234R*, T244R, F261C, F261L, F261S, Y321C, L328V, M329T, C325Y*, C325F*, C325W*, C325R*, R369S*, R369G*, V363I*, A365S, R369T*, R369K*, R369M UL51-A95V*	(216–219)
*Benzimidazole ribonucleosides* TCRB BDCRB	Terminase	HCMV	UL89-D344E UL56-Q204R	(208)
*Sulfonamides* BAY 35-5014 BAY 38-4766	Terminase, portal	HCMV	UL89-F142T, S143E, T144A, S237R, D358N, N353S, Y386H UL56-A662V UL104-F602I	(211)
WAY-150138	Portal	HSV-1	UL6-E121D, A618V, Q621R	(213)

^*a*^ Letermovir is FDA approved for prophylaxis of HCMV in adult and pediatric allogeneic hematopoietic stem cell transplant and high-risk kidney transplant recipients. Asterisks (*) indicate clinically observed resistance mutations. Benzimidazole ribonucleoside inhibitors, sulfonamide inhibitors, and WAY-150138 are pre-clinical and are not undergoing further clinical development.

Letermovir (AIC246) is the first and only FDA-approved small molecule inhibitor that targets viral genome packaging. Letermovir was identified by high-throughput screening and developed through hit-to-lead optimization and structure-activity relationship studies by AiCuris (215, 220). HCMV-infected cells treated with letermovir accumulate uncleaved concatemeric dsDNA and form significantly fewer mature C-capsids containing packaged dsDNA (221). Letermovir was licensed to Merck in 2012 and is currently approved for prophylaxis of HCMV in adult and pediatric allogeneic hematopoietic stem cell transplant and high-risk kidney transplant recipients (215, 222). Letermovir exhibits low nanomolar EC_50_ in HCMV cell models and is effective against strains that have developed resistance to existing antivirals (220). In patients, letermovir was shown to be non-inferior to valganciclovir, the standard of care treatment for high-risk kidney transplant recipients, with fewer toxicities observed, potentially because mammalian cells do not encode genes resembling the terminase (223).

Despite the high conservation of terminase subunits across the herpesviruses, letermovir is highly specific for HCMV, with no substantial activity against other herpesviruses, including HSV-1, VZV, and EBV (224). The precise mechanism of action for letermovir remains unknown. Clinically observed resistance mutations have emerged in terminase subunits UL56 and UL51 rather than UL89 (TerL), suggestive of a mechanism more complex than competitive inhibition of ATPase or endonuclease activity (216, 217) (Fig. 3; Table 3). *In silico* analysis of the HCMV terminase identified a putative binding cavity for letermovir in UL56 that is reduced in letermovir-resistant variants, and *in vitro* cell assays demonstrate that letermovir does not inhibit interactions between terminase subunits (225, 226). It is possible that letermovir may disrupt assembly of the oligomeric form of the terminase, allosterically inhibit enzymatic activity, or disrupt interaction with terminase-binding partners (i.e., portal).

The diversity of drug targets and putative binding sites for packaging inhibitors (Fig. 3; Table 3) suggests that multiple stages of viral genome packaging can be targeted with small molecule therapeutics. The clinical success of letermovir emphasizes the importance of obtaining detailed mechanistic information about herpesvirus genome packaging to develop similar inhibitors for other herpesviruses.

## THE OUTLOOK: DEFINING THE MECHANISM OF VIRAL GENOME PACKAGING IN HERPESVIRUSES

Decades of elegant biophysical and genetic analysis have revealed the molecular underpinnings of an ancient strategy to package dsDNA genomes. Reconstitution of a herpesvirus *in vitro* packaging system, along with high-resolution structures of capsids in the process of being packaged (Table 4), are critical next steps for further mechanistic dissection. Below, we highlight remaining open questions about the herpesvirus morphogenetic pathway.

**TABLE 4:** Structures of packaging components^*a*^

Component	Caudoviruses					Herpesviruses		
	Φ29	HK97	P22	λ	T4	HSV-1	HCMV	KSHV
Empty capsid/procapsid	6QVK	1IF0	8I1T 8I1V	7VI9	7ZRT	5ZAP		
Portal	6QX7	8FQL	8TVU	8XOU	6UZC	9OP5	8HEX	6PPI
TerL	5HD9 7CNB	6Z6D	4IEE	7LW0	3EZK	6M5S	3N4P (nuclease domain)	
TerS	N/A	6Z6E	3P9A	1J9I	3TXQ			
Other factors	1FOQ (pRNA)			2LSM (gpFI) 1OWG (IHF)	1E7D (gp49)	9ZLY (UL32)	9ZM2 (UL52)	6XF9 (ORF68)
Motor captured while packaging	7JQQ	EMD-16649 (map)						
Packaged capsid	1YXN 8F2O	1OHG	5UU5	7VII	5VF3	6CGR	5VKU	6B43

^*a*^ The Protein Data Bank codes for structural models are indicated.

### What principles of the viral genome packaging machinery are shared across the duplodnaviruses?

1. How does the herpesvirus terminase generate force? Are the subunits strictly coordinated? How does it adapt to nicks, gaps, and branches in newly replicated herpesvirus genomes?
2. Does the terminase package quickly to avoid cellular defenses, or does it lack the temporal constraints imposed by bacterial cell division? Is it possible to package dsDNA into capsids faster than 2,000 bp/s?
3. Why did herpesviruses evolve a three-component terminase complex?
4. What is the functional oligomeric state of the herpesvirus terminase, and does this differ from the pentameric bacteriophage terminases?
5. What is the role of the herpesvirus packaging accessory factor (HSV-1 UL32)?

### How is packaging regulated during herpesvirus infection?

1. How are expression and assembly of the terminase complex coordinated, and how is terminase activity (particularly endonuclease activity) regulated to prevent premature or off-target dsDNA cleavage?
2. How does the physical separation of capsid expression and assembly/packaging into distinct subcellular compartments impact regulation?
3. How are herpesvirus *pac* sites selected?
4. How is packaging coordinated with viral genome replication and late gene transcription?
5. How does letermovir inhibit the HCMV terminase, and can its inhibitory principles be applied to other herpesvirus terminases?

## AFTER EPUB

*[This article was published on 6 July 2026 with a preprint cited as reference 46. The reference was updated in the current version, posted on 24 August 2026.]*
